# Religion and Medicine

**Published:** 1909-01-02

**Authors:** Edwin Ash

**Affiliations:** 30 Harley Street, W.


### Editor's Letter-Box.

**Our correspondents are reminded that prolixity is a great bar to publication, and that brevity of style and conciseness of statement greatly facilitate early insertion.**

### RELIGION AND MEDICINE.

*To the Editor of* The Hospital.

Sir,—I am sure that a good many members of the medical profession besides myself have been glad to read your remarks upon the medical *rôle* lately assumed by certain clerical individuals. Personally, I feel confident that any cures that happen to be brought about by any of the various systems of religious healing that are now enjoying a wide vogue, are most certainly due to the workings of that important influence, daily becoming more appreciated in our profession, which we are accustomed to call " suggestion."

This being so it follows that, as you pointed out in your leader last week, such " religious treatment " can only cure in functional cases, which, as a matter of fact, happen to include some of the most obscure forms of illness with which we have to deal, disorders which frequently tax severely the diagnostic powers of highly-trained specialists. It is obvious, then, that lay practitioners of these pseudo-religious, pseudo-scientific systems of treatment are taking upon themselves the grave responsibilities of life and death, considering that in many diseases the delay of even a few hours in securing the right treatment may prove fatal to the patient.

I know that certain schools of religious healing are stated to call in a physician to decide which cases are suitable for treatment by mental means; but I fail to see any justification for taking such cases out of the hands of recognised practitioners, who are nowadays rapidly becoming more and more educated to the uses of suggestion in treatment.

The one thing I cannot understand about the latest so-called religious movement in connection with the healing art is why it has not raised a greater and more effective storm of organised protest in the ranks of the medical profession.

I am, yours, etc.,

Edwin Ash.

30 Harley Street, W., December 28, 1908.

